# Explanatory Interactive Machine Learning: Establishing an Action Design Research Process for Machine Learning Projects

**DOI:** 10.1007/s12599-023-00806-x

**Published:** 2023-04-21

**Authors:** Nicolas Pfeuffer, Lorenz Baum, Wolfgang Stammer, Benjamin M. Abdel-Karim, Patrick Schramowski, Andreas M. Bucher, Christian Hügel, Gernot Rohde, Kristian Kersting, Oliver Hinz

**Affiliations:** 1 Information Systems and Information Management, Goethe University Frankfurt, Frankfurt am Main, Germany; 2 Machine Learning Group, Department of Computer Science, Technical University of Darmstadt, Darmstadt, Germany; 3 Diagnostic and Interventional Radiology, Center of Radiology, Hospital of the Goethe University Frankfurt, Frankfurt am Main, Germany; 4 Pneumology and Allergology, Center of Internal Medicine, Hospital of the Goethe University Frankfurt, Frankfurt am Main, Germany

**Keywords:** Action design research, Data science, Explainable artificial intelligence, Interactive machine learning, Pneumonia, Corona virus

## Abstract

The most promising standard machine learning methods can deliver highly accurate classification results, often outperforming standard white-box methods. However, it is hardly possible for humans to fully understand the rationale behind the black-box results, and thus, these powerful methods hamper the creation of new knowledge on the part of humans and the broader acceptance of this technology. Explainable Artificial Intelligence attempts to overcome this problem by making the results more interpretable, while Interactive Machine Learning integrates humans into the process of insight discovery. The paper builds on recent successes in combining these two cutting-edge technologies and proposes how Explanatory Interactive Machine Learning (XIL) is embedded in a generalizable Action Design Research (ADR) process – called XIL-ADR. This approach can be used to analyze data, inspect models, and iteratively improve them. The paper shows the application of this process using the diagnosis of viral pneumonia, e.g., Covid-19, as an illustrative example. By these means, the paper also illustrates how XIL-ADR can help identify shortcomings of standard machine learning projects, gain new insights on the part of the human user, and thereby can help to unlock the full potential of AI-based systems for organizations and research.

## Introduction

Increasingly, it is becoming apparent that Interactive Machine Learning (IML), i.e., the integration of user feedback into a Machine Learning (ML) process to modify an ML model (e.g., Amershi et al. 2015), may play a leading role in shaping Artificial Intelligence (AI) and in particular ML-based systems for effective use in organizations. To realize their full potential, AI systems must become capable of communicating and collaborating with, learning from, and teaching their users. Finding the right ways to induce learning in human-machine interaction is required to unlock many scientific and commercial opportunities in AI (Teso and Hinz 2020).

Challenges to thorough understanding, potential learning from AI-based Systems, and effective organizational use result from the lack of system transparency (e.g., Rai 2020), which is often accompanied by the uncertainty of whether a system is biased. In the past five years, various scholars have shown the potential adverse effects of algorithmic biases on human decision-making (e.g., Lambrecht and Tucker 2019). Outcries demanding transparency and accountability have become louder and, as such, have found their way into legislation (e.g., Casey et al. 2019). Hence, there is an urgent need in many industries to fulfill such regulatory requirements for AI-based Systems (e.g., Sorantin et al. 2022; Casey et al. 2019). By now, many organizations are aware of this problem yet face a challenge in finding appropriate ways to deal with biases and erroneous systems (e.g., Holstein et al. 2019).

Researchers and practitioners have proposed various software engineering approaches that offer best practices for data science projects (e.g., Amershi et al. 2019; Wang et al. 2019). Many of these methods propose not a linear development process but a process that includes recursions to previous stages when necessary. Especially during model evaluation, data scientists need to reflect on the intermediate results of their work by, e.g., inspecting metrics and predictions and possibly rearranging their analytical workflow and data (e.g., Amershi et al. 2019). In this regard, research on data science collaboration (e.g., Zhang et al. 2020) suggests that – rather than having only data scientists working on their own – the inclusion of various kinds of users in the development process is beneficial to reducing algorithmic biases and increasing domain correctness.

Human-in-the-loop concepts (e.g., Grønsund and Aanestad 2020) and especially IML (e.g., Amershi et al. 2015; Abdel-Karim et al. 2020) constitute one possibility to include non-technical users in a data science process. Although human-in-the-loop methods have proven successful in improving various systems (e.g., Amershi et al. 2015) by including different kinds of users (e.g., Kulesza et al. 2015), users often are lost without appropriate explanations and thus do not know how to interpret and alter a system’s result appropriately (e.g., Dudley and Kristensson 2018, p. 24).

Schramowski et al. (2020) address this problem by including domain experts in a generic explanatory interactive machine learning (XIL) (Teso and Kersting 2019) process. While data scientists are usually aware of apparently wrong system attributions and can fix them on their own, XIL and the input of the domain expert can help identify and even correct predictors which only appear to be highly performant but are based on incorrect inferences.

There is an ongoing discussion on how interpretability, explainability, and transparency are related to each other and whether explanations (see Gregor and Benbasat 1999) help to better understand AI-based systems (e.g., Rudin 2019; Linardatos et al. 2020). Although both explainable and interpretable AI aim to bring more transparency to the application of ML, usually the former aims to do so by employing additional models (e.g., Ribeiro et al. 2016) that explain the ML model in focus (e.g., Deep Learning models, LeCun et al. 2015), while interpretable AI aims to use more lightweight, inherently interpretable ML models (e.g., GANs, Caruana et al. 2015). Rudin (2019) presents arguments that explanations of black-box models cannot provide the necessary insights to fully understand how such models arrive at their predictions (but have other benefits). This separates inherently interpretable models from ‘explained’ black-box models, which, on the one hand, offer benefits by usually providing greater modeling capacities, while the use of interpretable models, on the other hand, often requires less expertise (Rudin 2019, p. 206, pp. 208–210). Nevertheless, although the two streams appear to be in conflict, both share the common goal of providing insight into ML models. Hence, we use a more universal term of explainability for this work, as explanations provide insights that increase transparency and user acceptance of a system and help with the transfer of knowledge (Gregor and Benbasat 1999). Explanations support an analytical interpretation of black-box model results (despite them not being inherently interpretable).

Because XIL is a powerful yet flexible approach to interpreting and rectifying even systems with strong black-box characteristics, it may be a promising basis to leverage the full potential of AI-based systems for organizations. However, to do so, XIL needs to be embedded in a more generalizable approach to ease its applicability for machine learning projects in organizations and research alike.

Senior scholars have already recognized the need to extend existing methodologies such as action research (e.g., Maass et al. 2018) and see the potential of such extensions and improvements as valuable “*contributions to the IS community*” (Baskerville et al. 2018, p. 365). Furthermore, scholars theorizing on future problems caused by biased ML systems demand that IS should tackle the challenge of mitigating biases in ML systems (e.g., Kane et al. 2021, p. 375). In their investigation of the pitfalls of the strong reliance on ground truth values in AI, Lebovitz et al. (2021) emphasize the need to pay respect to ‘know-how’ measures (i.e., expert knowledge and knowledge that explains how an AI makes its decisions). It is, therefore, not sufficient to only rely on seemingly objective ‘know-what’ measures (i.e., ground-truth, performance metrics such as accuracy) (Lebovitz et al. 2021, pp. 1516–1517). These works imply the need for dedicated methods that enable a participatory, interactive, and explanatory workflow, honing problematic areas with great attention, diligence, and human expertise. The current paucity of applicable Information Systems Development Methods (ISDM) with foci on participatory feedback of users and the elimination of potential bias within machine learning projects thus suggests that we need to engineer suitable methods for this case.

We engage in situational method engineering (e.g., Goldkuhl and Karlsson 2020) and propose an ISDM that embeds cutting-edge technology, namely Explainable Artificial Intelligence (XAI) and Interactive Machine Learning (IML), referred to as Explainable Interactive Machine Learning (XIL) in the established framework of Action Design Research (ADR) (e.g., Mullarkey and Hevner 2019). The resulting XIL-ADR methodology positions itself as an implementation-centered ADR methodology along the elaborated ADR process that puts humans and machines into a loop which aims to remedy potential biases in an AI-based system and to generate novel insights on the side of the human user.

The proposed cyclic XIL-ADR process is generic and transferrable to other domains. In particular, it provides a development-focused organizational frame that is not limited to certain data types, specific algorithms for the explanations, ML methods, or the IML part. XIL-ADR certainly touches a truly interdisciplinary research field with connections to Computer Science, Learning and Knowledge Discovery on the human side, and domain knowledge from the application area.

To highlight the benefits of XIL-ADR, this paper uses a healthcare setting and presents an illustrative study conducted with medical experts from a leading university hospital. We exemplify our proposed approach in this healthcare scenario and show how XIL-ADR can help build an innovative computer vision system to efficiently (in terms of model performance) predict viral pneumonia. In particular, we will outline how XIL-ADR can help arrive at more meaningful models and, at the same time, help identify interesting patterns in the data that radiologists and pneumologists should pay attention to. The illustrative case study also reveals problems that, using standard approaches without participatory human-machine feedback, would be recognized much later, i.e., when the developed AI-based system fails in practice. Thus, the approach has two advantages: (1) it helps to create more dependable models, as it enables users to recognize intricate data and architecture problems early in the project, and (2) it simultaneously allows experts to reflect on the existing knowledge base by opening the otherwise black-box process of many Deep Learning (DL) algorithms and by this means may arrive at new insights and learning.

This paper proceeds as follows: First, we will introduce and conceptualize an ideal XIL-ADR process. We will do so by first outlining relevant research on IML, referencing XIL, embedding XIL in ADR, and then presenting how to structure ML projects for XIL-ADR. Next, we will briefly introduce our illustrative case of an AI-based diagnosis system for viral pneumonia, through which we aim to highlight how to apply XIL-ADR. We first introduce the reader to the problem to solve, previous research in computer vision, Covid-19, and the technical methods. This introduction also informs the first stage (*Diagnosis*) in our XIL-ADR project. For stage two (*Design*), we will give a brief overview of the technological methods we used, i.e., DL, XAI, the loss function for the penalization of the model, and the method for XAI visualization. What follows is the evaluation of the *Implementation* stage using the XIL-ADR methodology: A detailed description of the case study conducted together with pneumologists, radiologists, computer scientists, and information systems researchers is presented, which describes every step and intermediate artifact of the cyclic XIL-ADR implementation process in three subsequent cycles. Lastly, we discuss XIL-ADR considering the presented case and outline directions for future research and practice.

## Conceptualizing XIL-ADR

In this section, we engage in method engineering (e.g., Goldkuhl and Karlsson 2020) and embed XIL into the ADR framework. For this, we follow the “Method Engineering as Design Science” (ME-DS) process by Goldkuhl and Karlsson (2020) and develop a “*Category 4*” Information Systems Development Method (a “*Scholar generated ISDM targeted for business practice & scholars*”) (Goldkuhl and Karlsson 2020, pp. 1245–1246). The first activity is identifying the problem and motivating the development of XIL-ADR, which has already been dealt with in the previous section. The next step, theorizing the ISDM and engineering the method, follows now by briefly introducing the reader to relevant work on combining XAI and IML as well as to XIL itself. We describe how we can integrate XIL into the elaborated ADR process (Mullarkey and Hevner 2019) to engineer a novel methodology called XIL-ADR and point out the differences from classical ADR. We complete this method engineering activity by describing the different activities of the XIL-ADR cycle and how machine-learning projects may benefit from conducting XIL-ADR. For the last two activities of the ME-DS process, which are demonstrating and evaluating the proposed ISDM, we use our healthcare case presented in the subsequent sections. Hence, this paper aims at the demonstration and ‘communication’ (in terms of Design Science) of XIL-ADR (Goldkuhl and Karlsson 2020, p. 1250; Hevner et al. 2004).

### Related Research on Interactive Machine Learning (IML)

Scholars discovered the benefits of including non-technical users in IML approaches a while ago (e.g., Ware et al. 2001). Since these first steps in the domain of IML, research and interest in IML have increased exponentially (e.g., Dudley and Kristensson 2018). Amershi et al. (2015) emphasized the benefits of IML over Active Learning (AL), namely, that the user adopts an active role in the IML process, in contrast to the passive role of the “Oracle” in AL (Amershi et al. 2015, p. 109). As different authors note, however, non-technical users may not always be able to reason from system outputs and thus may have difficulties altering AI models for the better (e.g., Dudley and Kristensson 2018, p. 24). In the quest for greater user interpretability, various scholars concluded that equipping IML systems with an XAI component leads to not only an enriching experience but also better systems (e.g., Amershi et al. 2015, p. 111). For example, Kulesza et al. (2015) propose “Explanatory Debugging” as an effective methodology for empowering end users to not only interpret the results of ML-based systems through explanations but also give them the means to improve them interactively.

Teso and Kersting (2019), however, criticize prior approaches that have tried to combine XAI and IML as these considered the black-box characteristics of typical ML-based systems insufficiently by centering their methodologies too much around white-box methods. As an answer to the pressing problem of enriching not only white-box models but also black-box models with XAI and IML, they introduced their generalizable “Explanatory Interactive Machine Learning” (XIL) methodology, which was for the first time effectively instantiated by Schramowski et al. (2020) for DL models.

### Explanatory Interactive Machine Learning (XIL)

**Fig. 1:**
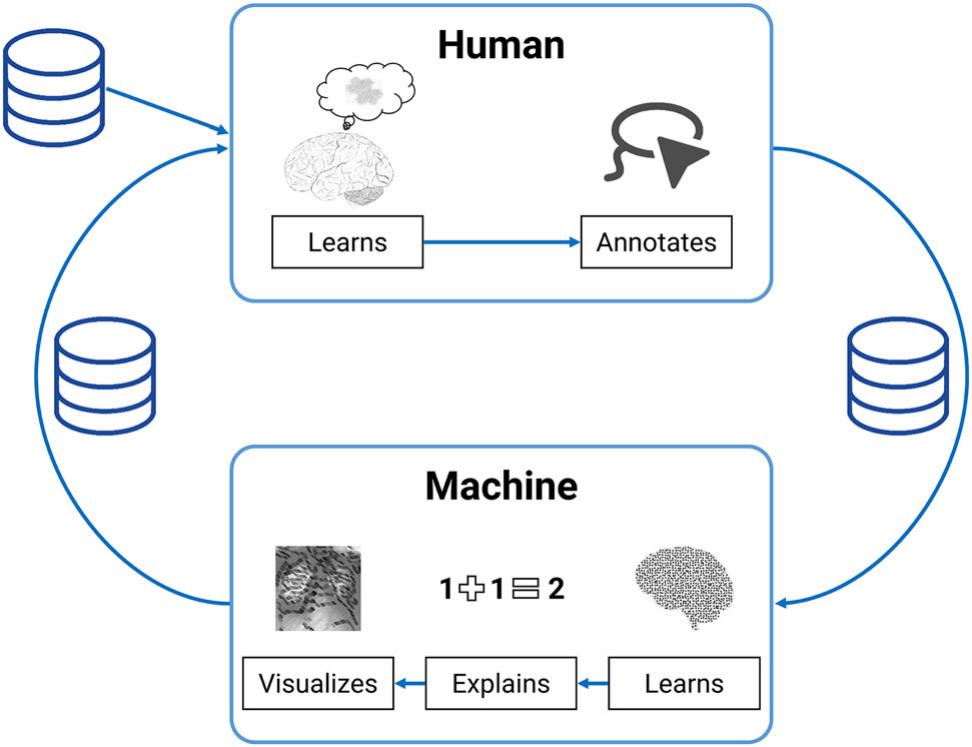
Explanatory interactive machine learning cycle

To close existing gaps and remedy the frequently insufficient connection of XAI and IML, XIL augments the iterative nature of IML with XAI such that the user interacts with the algorithmic explanations during the iterative training loops (see Fig. 1). In their paper, Schramowski et al. define XIL as follows (Schramowski et al. 2020, p. 478):

“*In XIL, a learner can interactively query the user (or some other information source) to obtain the desired outputs of the data points. The interaction takes the following form. At each step, the learner considers a data point (labeled or unlabeled), predicts a label, and provides explanations of its prediction. The user responds by correcting the learner if necessary, providing a slightly improved* – *but not necessarily optimal* – *feedback to the learner.*”

This process not only vividly combines the methods of XAI and IML but also allows the user to adjust labels in the training process. Figure 1 illustrates that based on the data and learned predictions, the Machine, i.e., the *learner*, produces and visualizes explanations that get presented to the Human, i.e., the *user*, who might then correct predictions and influence the machine’s learning process by annotating data, e.g., by pointing the machine toward features in the data to look at. Beyond this, by analyzing the explanations, the user can uncover incorrect algorithmic behavior if the XAI indicates a wrong explanation for a correct prediction. This fact provides the potential for uncovering and rectifying exceptionally intricate problems of ML through XIL (e.g., Schramowski et al. 2020, pp. 477–478). On the human side, this process also facilitates learning, e.g., regarding the data or model’s reasoning.

Though Teso and Kersting (2019) are proposing a XIL algorithm called ‘*CAIPI*’ (Teso and Kersting 2019, p. 241; Schramowski et al. 2020, p. 478) for pursuing explainable interactive machine learning, which we will also use in our medical evaluation case, this method is a generic process and therefore not restricted to specific methods for XAI or types of machine learning models. Instead, with XIL, various models are possible, i.e., black box and white box models (Schramowski et al. 2020, pp. 478–479), and explanations can and should be specifically selected for the ML task and type of users (e.g., Evans et al. 2022) involved in the XIL process (e.g., Holzinger and Müller 2021; Teso and Kersting 2019).

### Embedding XIL in the Elaborated ADR Process

Recent research on Machine Learning Systems finds that industry practitioners are yearning for structured methodologies for assessing systems regarding potential biases and how to fix them subsequently (e.g., Holstein et al. 2019). Various methodologies for combining IML with XAI have emerged (e.g., Kulesza et al. 2015), yet little emphasis has been put on how these promising methodologies could be integrated into organizational settings.

**Fig. 2:**
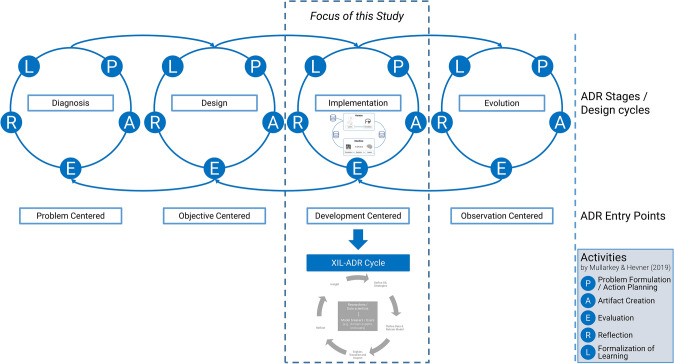
Integration of XIL in the elaborated ADR Process as XIL-ADR, adapted from Mullarkey and Hevner (2019)

ADR has always seen the practical evaluation of IT-based phenomena as substantial to generating theoretical, methodological, technical, and organizational insights (e.g., Mullarkey and Hevner 2019). In an analysis of ADR in practice, Haj-Bolouri et al. (2018) criticize that most activities in classical ADR (i.e., Sein et al. 2011) terminology may be found in the building stages, less so in the intervention and evaluation stages, and least in the reflection and learning stages. As especially the evaluation of ML-based systems needs to be handled with care and requires attention, this finding is problematic for conducting ML projects with classical ADR approaches (e.g., Sein et al. 2011; Mullarkey and Hevner 2019). This problem leads to the need to adapt ADR for explainable interactive machine learning. From the IS lens, much speaks for XIL to be integrated into ADR as an “*IT-Dominant*” (Sein et al. 2011, p. 42) ADR approach focusing on the *Implementation* (e.g., Mullarkey and Hevner 2019) (see stages in Fig. 2).

Starting from the elaborated ADR Process by Mullarkey and Hevner (2019), XIL-ADR incorporates changes to the *Implementation* stage. Figure 2 shows that the *Diagnosis*, *Design*, *Evolution* stages of the process stay untouched, while the cyclic process within the *Implementation* stage is adapted by XIL-ADR to better suit the requirements and specialties of machine learning projects along the lines of XIL. Each of these stages consists of five activities (*P*, *A*, *E*, *R*, *L*) (Mullarkey and Hevner 2019, pp. 3–4). Overall, also with XIL-ADR embedded into ADR, the stages’ goals stay the same: The *Diagnosis* stage focuses on identifying the business problem and helps the researcher comprehend the project domain and the practitioner, i.e., model breaker or user, get an overview of the state-of-the-art (Mullarkey and Hevner 2019, p. 4) of, for example, the ML methods applicable. Within the *Design* stage, design principles are developed to tackle the identified problem, and the *Implementation* stage is prepared. Next, the *Implementation* creates the artifact.

“*Typical artefacts abstracted and evaluated in the ADR Implementation cycle include systems, algorithms, programmes, databases, and processes*”

(Mullarkey and Hevner 2019, p. 5); in our case, the process of using XIL to come to an AI-based solution to the problem. The main difference with XIL-ADR concerns the activities of the *Implementation* stage, which are elaborated in the following subsection. Finally, the *Evolution* stage allows for continuous adaptation of the artifact, i.e., the AI system, to the environment as it progresses. This stage becomes necessary with technology, data, or model-specific characteristics changing over time. Here, depending on the type of evolution, cycles with XIL-ADR-specific activities (from the implementation stage) or with regular ADR activities may or may not be desired. In ADR as well as in XIL-ADR, forward and backward jumps between these stages are possible and contribute to the flexibility of these approaches (note the arrows in Fig. 2) (e.g., Mullarkey and Hevner 2019, p. 5).

With the focus of XIL-ADR on the *Implementation* stage, two main differences between classical ADR (i.e., Sein et al. 2011; Mullarkey and Hevner 2019) for implementations and XIL-ADR emerge. First, in classical ADR, the *BIE (Building-Intervention-Evaluation)*-stage is a central module for achieving organizational innovation, change, and success of the ADR project (Sein et al. 2011). Interventions can be anything that addresses the organizational problem of concern, i.e., design principles or a full-blown technical artifact (e.g., Sein et al. 2011, p. 42). As such, we can see interventions in classical ADR directed toward the organizational context. XIL-ADR provides a different perspective toward interventions (called *XIL Strategies*) in that it focuses on technical interventions that lead to direct effects on the AI-based system, based on the evaluations. Following this view, XIL-ADR should instead resemble a *Building-Evaluation-Intervention (BEI)* process scheme, as opposed to the standard *BIE* scheme of classical ADR approaches (e.g., Sein et al. 2011, p. 42) (refer to the following subsection). More importantly, the *Building* stage in XIL-ADR (called *Refine Data & Retrain Model* activity) can be viewed as an operationalization of the interventions taking place in XIL-ADR: It is merely an action based on the novel engineering strategies that emerged from the solid evaluations and reflections of previous design cycles. In emphasizing the importance of interventions in XIL-ADR, we are thus in line with the propositions of Mullarkey and Hevner (2019, p. 8).

One of the more intricate differences between classical ADR (i.e., Sein et al. 2011; Mullarkey and Hevner 2019) and XIL-ADR is that XIL-ADR also introduces the concept of cyclic renewal of the artifact’s basis. While in standardized ADR projects with classical IT tools, system prototypes can be built based on defined design guidelines, system syntax, and rulesets, projects involving ML-based systems face fundamentally different circumstances (e.g., Teodorescu et al. 2021, p. 1494). Because modern ML-based systems, such as DL systems, learn from data and build a model based on selected statistical and often non-linear methods, they provide promising solutions to fuzzy sets of problems (e.g., LeCun et al. 2015). Due to this very feature, developers and system users can often hardly recognize intricate data and architecture problems in the pilot stages of development (e.g., Schramowski et al. 2020). Nevertheless, when ML models fail due to the data foundation (e.g., false ground-truth labels, confounders in training data, cascading data errors, see Sambasivan et al. 2021) or due to unfitness of the model’s basic architecture, appropriate responses must be made. As such, XIL-ADR integrates this potential for a cyclic renewal of the artifact’s basis into its method (which, without this, required a jump to the *Design* stage).

In summary, XIL-ADR provides an iterative process aimed at improving ML-based systems and at eliminating potential errors and biases. In contrast to conventional IML, the integration of explanations and visualizations enables participants to interpret, reflect on and learn from the system’s reasoning about the results. Up to this point, IS research has not contributed much to illuminate the benefits of IML (e.g., Grønsund and Aanestad 2020) for data science in organizational settings, yet it emphasizes the importance of system explanations for human-in-the-loop configurations. In this regard, XIL-ADR can enable the engineering of high-performance, less biased, and insightful ML models for practice and presents the opportunity for more profound insights into human-machine collaborations (see also Fig. 1).

### Implementing Machine Learning Projects with XIL-ADR

**Fig. 3:**
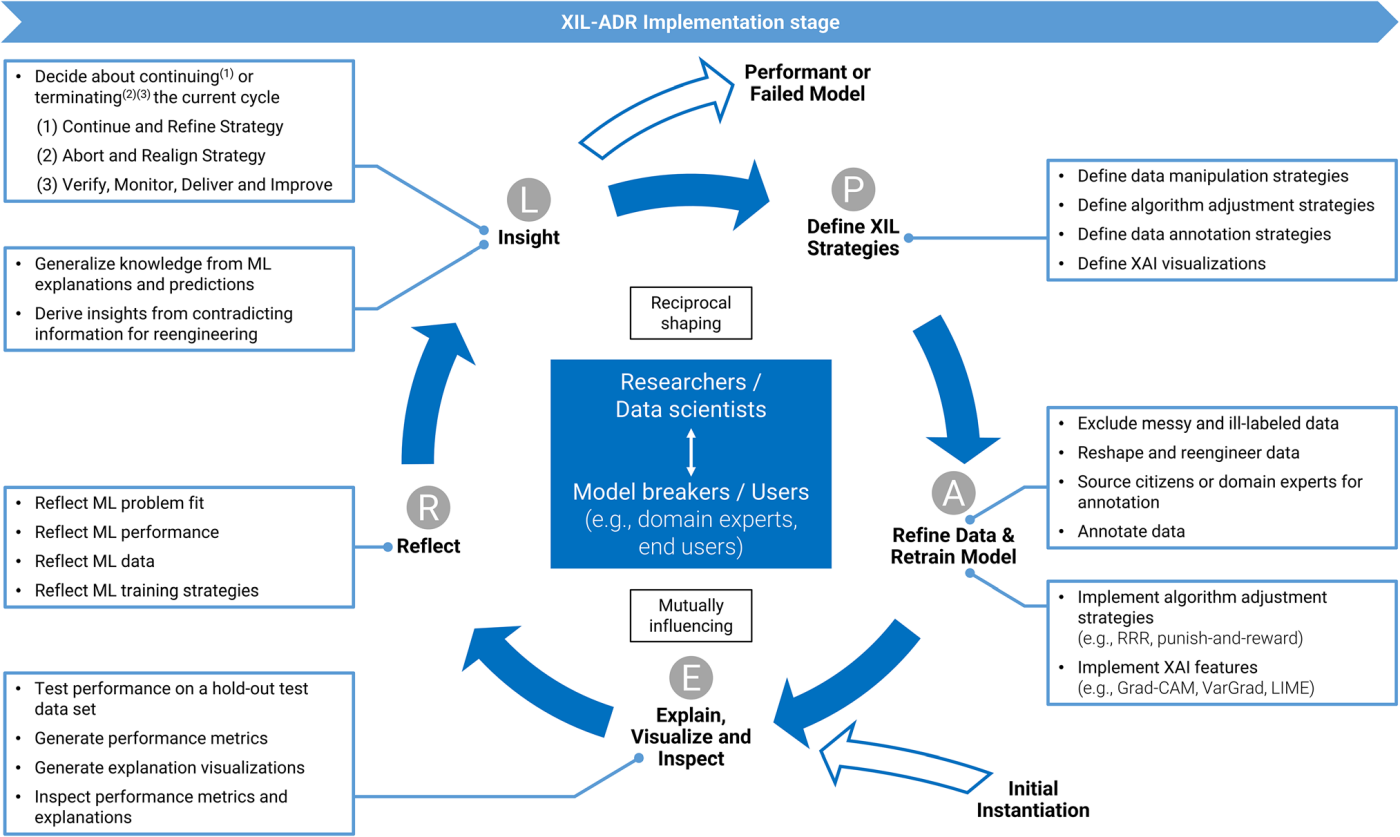
Activities of the XIL-ADR Implementation cycle embedded in the elaborated ADR Process, adapted from Mullarkey and Hevner (2019)

To enable research and practice to adopt XIL-ADR for machine learning projects, we present it in a generalized manner (see Fig. 3). In classical ADR (i.e., Sein et al. 2011; Mullarkey and Hevner 2019), we find two main parties that reciprocally shape and mutually influence a development cycle and each other (e.g., Mullarkey and Hevner 2019; Sein et al. 2011). Also, in XIL-ADR, on a high level, we find two groups of actors: Data scientists, who provide a technical perspective on the matter of development, and domain experts, who provide a non-technical but domain perspective. Together they are called the development team. As Zhang et al. (2020) put forward, including various kinds of users directly in the development of ML projects not only opens up the potential for closer domain fit but can also result in less biased systems (Zhang et al. 2020, p. 11). We recommend including different kinds of users and thus a highly diversified team in XIL-ADR processes, since we believe that the orthogonal picture that may form in group-discussions could greatly benefit the emergence of better models in machine learning projects (e.g., Lave and Wenger 1991). Especially valuable in this regard are so-called “*model breakers*” (Hong et al. 2020, p. 8), who may contribute strongly to improving the model by means of critical inspection and the application of their domain knowledge. Such “*model breakers*” may come in the form of domain experts, auditors, or product managers (Hong et al. 2020, p. 8). For example, depending on their background, model breakers may uncover semantic problems from a knowledge domain perspective (domain experts) in addition to unfulfilled regulatory or legal requirements (auditors).

The cyclic process of XIL-ADR consists of five activities executed in cycles. In the elaborated ADR process, Mullarkey and Hevner (2019) call these activities “Problem Formulation/Action Planning (P)”, “Artifact Creation (A)”, “Evaluation (E)”, “Reflection (R)”, and “Formalization of Learning (L)” (Mullarkey and Hevner 2019, pp. 3–4). To better fit the *Implementation* process for AI systems and guide the application of the proposed process, in XIL-ADR, the activities have different purposes and require different actions. Also, the activities’ naming is adapted to be more descriptive regarding the performed actions (see the letters in Fig. 3 for an idea about how the activities were renamed).

A first prototype is needed to enter the XIL-ADR cycle. Hence, the process starts with an *Initial Instantiation* of a model. Commonly, engineering knowledge largely informs the *Initial Instantiation*, and it is thus missing potentially important pieces of domain knowledge. As such, the data and model will be primarily prepared along known software engineering processes (e.g., Amershi et al. 2019) and will provide an ingenious starting point for the upcoming XIL-ADR cycles.

Following Fig. 3, the next activity in XIL-ADR focuses on actions around *Explaining, Visualizing and Inspecting*. In this step, transparency-based evaluation criteria are needed to come to a transparency-based evaluation report as the intermediate artifact of this activity. Therefore, the team conducts performance tests along known metrics (e.g., Accuracy, Precision, Recall) to get an overall feeling about the model’s performance. However, these metrics must be accompanied by XAI (either through metrics or visualizations) that contextualize model performance either by explaining the prediction (local XAI), overall model workings (global XAI), or ideally both (Bauer et al. 2021). As Hong et al. (2020) contend, it is crucial to design XAI features according to the different users’ needs, such that these users can effectively engage in the model inspection. With this, they are in line with the results of Evans et al. (2022), who compared different XAI techniques in a pathology case (Evans et al. 2022, p. 293).

A pivotal step in the XIL-ADR cycle is the *Reflect* activity. Data scientists often focus on technical rationality (e.g., Neumann 2000, pp. 404–406) and overlook missing domain logic and potential domain-specific implementation requirements, which may frequently only be realized at the reflection step (Sambasivan et al. 2021). Thus, the team has to critically reflect on the fit between the model and the problem while considering the domain and business context. For the reflection activity, the team consisting of data scientists and domain experts must also deliberate on the model’s performance in light of the explanations. Additionally, this step includes a critical reflection of the underlying data, as well as a reflection of training and testing strategies. Following the investigations of Lebovitz et al. (2021), project teams should use the available XAI as an opportunity to thoroughly scrutinize the AI model, even questioning the validity of its ground truth data. This way, there is a higher potential for uncovering model flaws pertaining to the data (e.g., low model performance, wrong features prioritized, data not providing the necessary information, or insufficient and inconsistent quality).

The realization of *Insights* follows after the group reflections. Such insights may be of an engineering nature, i.e., the realization of novel implementation requirements. Besides, especially with growing model maturity, there is also the potential for an extension of the knowledge base by insights generated through the inspection of explanations (e.g., Teso and Hinz 2020). In this activity, it is important to view the models as malleable and not solely focus on technical performance metrics since each participant may value these metrics differently, which can hinder the progress in the iterative model development (e.g., Passi and Jackson 2018, p. 13). Furthermore, in line with Passi and Jackson (2018, p. 13), the *Insights* activity needs to discursively balance situations in which insights warrant new implementation requirements or insights pose potential extensions to the body of knowledge. This activity is also the point of each cycle where the development team has to decide about possibly exiting the design cycle. Details on this decision process follow after completing the description of the XIL-ADR cycle.

As the last activity in XIL-ADR, *Define XIL Strategies* formalizes previously captured implementation requirements into strategies for data manipulation, algorithm adjustment, data annotation, and XAI visualizations. Apart from distinctions that must be made in data and algorithm work based on the machine learning task, the data annotation strategy requires special attention. In general, annotations may critically improve and speed up model work (e.g., Amershi et al. 2015). Nevertheless, there is a risk that annotations for large datasets may be costly when created by experts or may not warrant the expected quality of results when created by citizen scientists (e.g., Weinhardt et al. 2020, p. 275) or crowd workers. Depending on the type of ML task or data and annotation task, hybrid approaches where just parts of the data get annotated may be possible. Usually, after the *XIL Strategies* have been defined, XIL-ADR continues to iterate with a new cycle. XIL-ADR may also spawn sub-cycles if the definition of strategies proposes to do so (this will be shown in loops 3a and 3b of our case study). After the *Define XIL Strategies* activity, the strategies will be implemented in the first activity of the next loop (i.e., *Refine Data & Refine Model*).

A subsequent new XIL-ADR cycle starts with the *Refine Data & Refine Model* activity which is performed instead of the *Initial Instantiation*. In this activity, the development team implements the previously defined strategies. This involves work on the data basis and the AI model itself. Actions directed to the data include handling ill-labeled data, reshaping or reengineering data, and annotating data. The activity may also require the team to find domain experts or citizens for the possible annotation of the data. Actions directed to the model include adjusting the algorithm and implementing (new) XAI features to enable the team to better understand the model’s reasoning. After the *Refine Data & Refine Model* activity, the cycle continues as described and as Fig. 3 suggests with *Explaining, Visualizing and Inspecting*.

XIL-ADR has the limitation that the current cycle may only be exited after the *Insights* activity. Therefore, the development team has to decide how to proceed at the end of the *Insight* activity. In general, there are three distinct possibilities of outcomes of this decision process: (1) ‘*Continue and Refine Strategy*’, (2) ‘*Abort and Realign Strategy*’, and (3) ‘*Verify, Monitor, Deliver and Improve*’. Outcomes (2) and (3) lead to a *Performant or Failed Model* in Fig. 3.

For the first outcome, i.e., (1) *Continue and Refine Strategy*, the XIL-ADR cycle continues as usual with the *Define XIL Strategies* activity, and a new cycle starts subsequently. For this outcome, *Insights* must not reveal severe problems regarding the model or the data. The team must be satisfied with the model, which includes sufficient satisfaction from the domain experts’ side (face validity) and the performance measures looking promising. Additionally, there still needs to be the potential for improvements, with some doubts or open strategies remaining, requiring a subsequent *Implementation* cycle. When the Insight activity reports serious problems with the data or the AI model, the outcome will be (2) *Abort and Realign Strategy*. Criteria for this outcome are either low performance measures or the majority of domain experts rejecting the model. In this case, the team stops the process and has to make substantial considerations about how to proceed. Jumps to preceding stages, i.e., *Diagnosis* or *Design*, present potential solutions to this problem, but the development team must also consider the process’s complete cancellation – especially under economic constraints. Finally, the outcome can be (3) *Verify, Monitor, Deliver and Improve*. Indications for this include the model being effective in solving the problem, based on the performance measures, and the domain experts uniformly approving the system (face validity). The potential for further improvements has to appear very low, so that a continuation of the *Implementation* does not make sense from an economic or business perspective. In this case, the iterative XIL-ADR process may be promoted to further quality assessments, testing, and subsequent deployment cycles in practice, i.e., by continuing with the *Evolution* stage (see Fig. 2).

## Evaluation Case Overview

Guided by the “Method Engineering as Design Science” (Goldkuhl and Karlsson 2020) process, the following two sections serve as the demonstration and evaluation of the proposed ISDM method. To illustrate how we envision the application of XIL-ADR in research and practice (Goldkuhl and Karlsson 2020), we conducted an exemplary ADR case study. In the following, we provide an overview of our case study situated in healthcare. This case description also guides the reader along the first two of four stages in ADR as put forward by Mullarkey and Hevner (2019), i.e., the *Diagnosis* and *Design* stages (refer to the section “Embedding XIL in the Elaborated ADR Process” for an overview of the purposes of these stages). While the developments of current research on Computer Vision in Health IT informed the domain experts and the domain-specific research on Covid-19 informed the researchers in the *Diagnosis* stage, discussions with clinical personnel (i.e., pneumologists) on the application of ML-based systems in healthcare largely affected the *Design* stage. For the *Design* stage, we also introduce the data basis and the technical methods to prepare for the XIL-ADR-based *Implementation* stage in the subsequent section. The subsection on technical methods also includes (technical) insights from the *Implementation* cycles, e.g., about the XAI visualizations used. We chose this presentation approach to separate the technical results from the evaluation of the XIL-ADR method, which takes place in Sect. 4, “Evaluation Case Study”.

### Diagnosis Stage: Background of the Application Domain and Data

Pneumonia is an inflammatory condition of the lung and can be caused by bacteria, a virus, or, less commonly, by other microorganisms. Each year, pneumonia affects about 450 million people globally and results in about four million deaths (Ruuskanen et al. 2011). With the advent of the novel corona virus (SARS-CoV-2), we observe a surge in viral pneumonia cases worldwide. While the WHO has been reporting up to millions of new cases each day for the past two years, governments worldwide have taken drastic measures to relieve the strain the pandemic has on our health systems and medical staff (Lai et al. 2020; McCall 2020). Although the policy measures to “flatten the curve” have shown some effect (Gibney 2020), the race between scientific research to treat and contain the disease and the growing discontent of the population with the perceived limitations is still ongoing.

Pinpointing exact symptoms in contrast to conventional viral pneumonia and the correct treatment at each stage of the disease (Chen et al. 2020a) now appears to be more critical than ever. Nevertheless, several factors limit the efficiency of hospitals and medical practices and place a heavy burden on staff. Firstly, high medical expertise is often a scarce resource. Second, inexperienced specialists may have difficulty recognizing novel and unusual disease patterns in coronavirus-affected patients (e.g., Schubert et al. 2013). Thirdly, even with high expertise, medical doctors are (even in everyday situations) put under high time pressure, which challenges the extremely precise and quick decision-making required (e.g., ALQahtani et al. 2018; Tsiga et al. 2013), which may be necessary for adequate treatment and allocation of medical resources for viral pneumonia cases.

Therefore, primary concern in a pandemic is the efficient use of available personnel. Trained medical experts cannot easily be scaled to growing demand. Radiological imaging has been used as a primary screening tool in some countries and has proven essential for detecting, ruling out, and monitoring pulmonary infections with SARS-COV-2. Researchers have suggested automatic detection methods for several reasons: providing decision support, providing a quantitative measure for diagnostic purposes, and providing a predictive tool. Further, automatic detection and diagnostic assistance tools can help substitute for trained personnel in a region with low availability of medical human resources.

To make viral pneumonia easier to detect and relieve and to assist physicians in diagnosing and treating the disease, scholars have tried to create effective ML-based expert systems that can predict positive cases of Covid-19-infected patients from radiography (e.g., Shi et al. 2021). Several of these systems achieve high accuracy on the data available to the researchers (e.g., Wang et al. 2021). However, we should consider the actual usefulness of these systems in reality with the limitation in mind that the image data that these systems use might not provide a perfect, comparable, and uniform data basis, and therefore, the accuracy of these models may not hold in practical use (Shi et al. 2021, pp. 11–12). Furthermore, only very few of these systems (e.g., Wang et al. 2020) currently offer plausible explanations for their predictions, which, on the one hand, is a critical factor for the usefulness of such systems in practice and as a driver for trust in these systems. On the other hand, there is evidence that being able to interactively modify a system further increases trust, satisfaction, and thus efficient use of these systems (e.g., Dietvorst et al. 2018). Finally, in the case of clinicians or radiologists reading chest radiographs (CXR), this reader would be able to check and intervene quickly, adding a layer of safety to the diagnosis. With these aspects in mind, we expect that a system developed with the help of XIL-ADR that receives sufficient evaluation and adjustment by medical experts through an IML process and provides fine-granular explanations through XAI could not only be more useful in practice but may also lead to differentiated insights regarding patterns that may be indicative for Covid-19.

### Diagnosis Stage: Related Work in Computer Vision and Radiology

Typically, after first anamnesis and determining abnormalities within clinical metrics, one possible further examination is to have patients with suspected lung disease screened via radiography. A radiologist would then analyze CXR or CT images and provide a diagnosis based on the patterns shown by the medical images.

Unfortunately, in many regions, there are too few radiologists per hospital and even per country (Ekpo et al. 2015), such that not only the large throughput of CXR images causes enormous amounts of stress (McDonald et al. 2015), but also a large backlog of CXR images and diagnoses arises (Yates et al. 2018). For this reason, many countries have implemented the red dot or asterisk system (Berman et al. 1985), which allows radiographers with additional specialization and training to give a first technical assessment if an image contains abnormalities. Therefore, the clinicians requesting radiography receive indications of abnormalities faster than before. Different studies (e.g., Brealey et al. 2006; Ekpo et al. 2015) illustrate the value of red dot systems in speeding up diagnostics and patient treatment without significant sacrifices in accuracy.

Since the advent of DL, scholars in the area of computer vision and radiology alike have shown increased interest in developing ML-based approaches to red dot systems. Such systems may not only assist clinicians by speeding up diagnosis and treatment but also by facilitating repeatability and potential reproducibility of decisions and automated processing of substantial amounts of comparable datasets in case of case registries. Mei et al. (2020), for example, show that AI-based systems continuously improve and reach similar levels of sensitivity and specificity compared to specialists in their field.

In the area of viral pneumonia, many scholars have tried to create effective and efficient ML-based expert systems that can predict positive cases in patients from radiography (e.g., Shi et al. 2021). While many of these studies lack interaction with clinicians and only present engineering approaches, some studies that include an assessment of the system through interaction with clinicians have already shown promising results. Chen et al. (2020b), for example, implemented a Convolutional Neural Network (CNN), which was able to classify control patients with other diseases and patients with viral pneumonia caused by Covid-19 from computed tomography (CT) scans. In an experiment with an expert radiologist, they found that the radiologist required significantly less time reading CT images and detecting a patient with viral pneumonia (i.e., Covid-19-based pneumonia in this study) when receiving diagnostic aid by the CNN compared to reading the CT images only on his own.

As evidence shows and discussed by Kundu et al. (2020) and McCall (2020), these systems may speed up the diagnosis process for many diseases enormously when employed correctly. Currently, there is also some discussion on whether CXR or CT provides a better foundation for AI-assisted diagnosis. Nevertheless, many clinicians agree that especially portable X-ray generators are helpful for a faster and less complicated screening process (Jacobi et al. 2020).

We use the case of building a diagnosis system based on CXR, i.e., X-ray images with lower resolution than CT scans, as an illustrative example to implement the proposed XIL-ADR methodology. For this purpose, we integrate radiologists and pneumologists from a leading European university hospital into the XIL-ADR process. A primary goal of this process is to arrive at a system that (1) can deliver more differentiated results than a traditional ‘red-dot-system’-like AI-based system (i.e., not only flagging images with potential abnormalities (Yates et al. 2018), e.g., by providing some kind of confidence measure), (2) that is able to deliver explanations regarding its predictions, and (3) that delivers more reliable results than conventional, non-XIL trained AI-based systems, due to the fact that with XIL expert knowledge directly influences the results. By applying XIL-ADR, we show how we unravel problems with data and develop and shift XIL strategies to get closer to this goal with each development cycle.

### Design Stage: Data Basis

The development of such a system requires not only abundant computational resources but also suitable data. Fortunately, since the pandemic started, not only enormous amounts of studies have been conducted that focused on a better understanding of the disease and on finding therapeutic methods, drugs or vaccines, but also many initiatives have tried to improve the networking and exchange of data sources to facilitate the development of technical solutions to overcome the pandemic.^1^

We started developing our system based on the “COVID-19 Radiography Database” provided by Chowdhury et al. (2020).^2^ This publicly available database comprises radiographs of patients with suspected lung diseases caused by pathogens from various sources (e.g., Cohen et al. 2020) and won the Kaggle Dataset award. To date, many researchers reference or use this dataset for their studies (e.g., Apostolopoulos et al. 2020; Apostolopoulos and Mpesiana 2020; Ucar and Korkmaz 2020; Yamaç et al. 2021).

The X-ray images included in this database were initially taken during examinations and marked with the final diagnosis so that labels corresponding to the diagnosis are available for the radiographs. This process resulted in a database with labels for (1) healthy patients, (2) cases of pneumonia caused by Covid-19, and (3) cases of pneumonia caused by conventional viral pathogens. This foundation (see Table 1 for more details and sample data) enables us to build a classifier that may be able to distinguish efficiently between these three classes.

**Table 1:** Covid-19 Radiography Database provided by Chowdhury et al. (2020)

Types	(1) Normal	(2) Covid-19	(3) Viral Pneumonia
Count	1341	219	1345
Examples			

### Design to Implementation Stage: Technical Methods

As we mentioned in the theoretical background, we instantiate XIL-ADR in the development-centered *Implementation* stage of ADR (stage three, see Fig. 2). This section provides some insights into the technical methods needed for the specific XIL-ADR process (*Design* stage) and technical insights from the *Implementation* stage.

The proposed cyclic XIL-ADR process consists of innovative approaches from computer science. Deep Learning lays the foundation for the process. We open the black-box by using methods coming from the area of XAI. To allow humans to bring their expertise into the process, we use IML employing the *CAIPI* algorithm (Schramowski et al. 2020, p. 478) and then start the Deep Learning process again afterward. By combining these different approaches, it is possible to remedy the most pressing problems in ML. We will later exemplify the process and will share a few insights from the area of medical imaging.

#### Deep Learning

We decide to train a CNN on the X-ray dataset. CNNs are a category of models of DL. We use the AlexNet version in the PyTorch library from Facebook (Paszke et al. 2019) that has been pre-trained on ImageNet data (Deng et al. 2009). The decision to use the AlexNet architecture results from numerous preliminary studies with different network architectures, such as ResNet, SqueezeNet, and DenseNet. The AlexNet architecture is based on the work of Krizhevsky et al. (2012). In CNN literature, this well-known model has demonstrated its capability in the field of image classification.

This CNN model has eight weighted layers, consisting of five convolutional layers followed by three fully connected layers (Krizhevsky et al. 2012). We transformed the X-ray images to AlexNet input size and thus fed them into the network with a resolution of 224 × 224 pixels. In the context of our AlexNet parameterization, we use a global learning rate of 0.0001 for all layers. We implement RMSProp as the optimizer with an alpha of 0.99, epsilon of 1 × 10^−8^, a weight decay factor of 0, and a momentum of 0.09. Based on the PyTorch DL framework, we divide our dataset into a train, a validation, and a test set. We utilize the validation set for hyper-parameter tuning and the test set to determine the neural network’s performance. The test set contains unique X-rays sampled from the raw dataset, which are not in the train or validation dataset (Chowdhury et al. 2020). We use a random fraction of 50% from the source dataset for the training dataset. For the test and validation datasets, we randomly split the remaining set of X-rays again in fractions of 50%. We randomly rotated each image in the training set between − 15° and 15° relative to its stored position from the source dataset and randomly cropped and flipped it horizontally and vertically. We use the labels for each class label provided in the source dataset as ground truth. To improve image quality in the context of training, we make use of classical image normalization.

#### Explainable AI

As already mentioned, ML methods have a growing impact in more and more areas and applications, especially grounded in the latest advances in Deep Learning. Domain experts usually do not explicitly implement the decision process of such models. Instead, the machine itself learns it from the provided data. The result is that the user or domain expert loses the influence on how and why a machine is making its decisions. XAI tackles the latter one. Within XAI, one must distinguish between white-box machine learning algorithms that are inherently explainable (e.g., decision trees and Bayesian classifiers) and black-box models. Deep Learning models are commonly used black-box models. XAI methods (e.g., Lundberg and Lee 2017; Ribeiro et al. 2016; Selvaraju et al. 2017; Simonyan et al. 2014) are trying to open these black-box models to explain the learned decision process. On the other hand, interpretable models not only provide explainability and understandability but do this inherently, offering these benefits with the downside of giving up flexibility in the model choice (i.e., difficulties using DL) and increased effort in development (e.g., Rudin 2019, pp. 208–210). Here, an interesting approach is the design of interpretable Deep Learning architectures (Chen et al. 2019), which create an intermediate interpretable layer within the DL algorithm. The user can use this layer to understand the classification. With XAI, the resulting explanation can have different modalities; often, however, visual feature importance maps are provided, e.g., in the form of heatmaps. For medical images, like in our example case, they are well suited as easy-to-understand visual explanations that reflect the way of thinking of pneumologists and especially radiologists (Evans et al. 2022, pp. 282, 293) and serve an understanding of the underlying model, which is crucial for the use of XAI in XIL.

#### XIL

The algorithmic methodology applied in the context of XIL, i.e., *CAIPI*, can be regarded as model agnostic (e.g., Teso and Kersting 2019, p. 239). As outlined in Fig. 3 in the section “Implementing Machine Learning Projects with XIL-ADR”, we employ the XIL optimizer (i.e., *CAIPI* algorithm with “*right-for-the-right-reasons loss*” (RRR)) as proposed by Schramowski et al. (2020) as part of our strategy for algorithm adjustment. In this regard, the optimization function of XIL uses annotations (i.e., a binary mask generated from human-created annotations) to penalize the model using parts of an image that lie outside the annotations. In doing so, the XIL optimization function, i.e., *CAIPI*, is proposed in the paper of Schramowski et al. (2020) (see pp. 478–479 for more details on the underlying theorem) and adjusted from Ross et al. (2017). It helps the ML model to focus better on relevant regions by penalizing attributions outside annotated areas, i.e., the model learns to base its predictions on annotated regions while paying less attention to penalized areas.

**Fig. 4:**
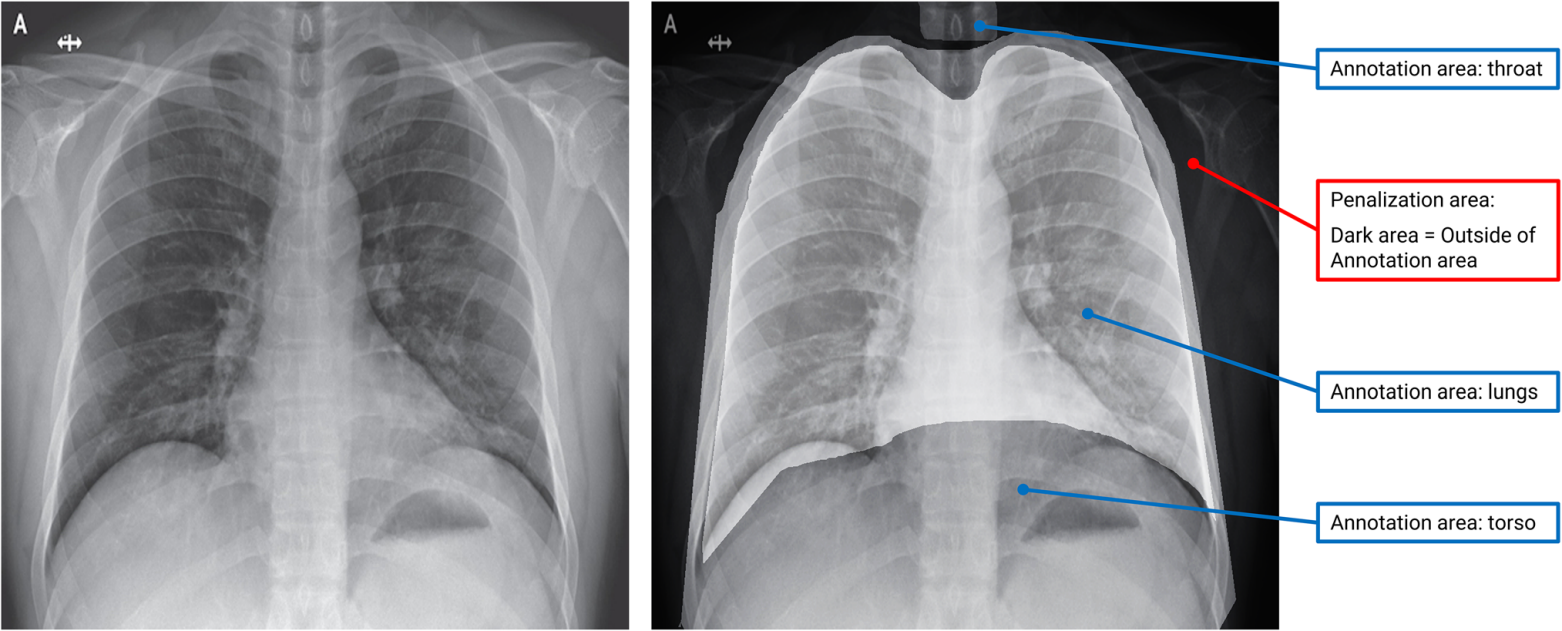
An exemplary depiction of how our XIL optimization strategy uses the annotations. The original image is on the left, and the same image is on the right with different annotation regions (highlighted in bright colors). Based on the optimization strategy, dark areas denote image regions, which the model should not focus on to make its predictions and lead to penalization in case it does (color figure online)

Figure 4 shows an exemplary depiction of how the XIL optimizer uses annotations for an image to influence algorithmic attribution. The picture on the right shows the same picture as on the left, with different annotated areas (highlighted for exemplary purposes in bright grey). Everything (i.e., every gradient/pixel) that lies outside these annotation masks is subject to penalization. This way, our model is directed toward attributing the relevant image features.

#### XAI Visualization

To visualize the explanations of the used neural network classifiers, we used two separate XAI methods with varying colormaps. For coarse image explanations, we applied the Grad-CAM method (Selvaraju et al. 2017), representing a trade-off between spatial information and higher-level visual constructs. Grad-CAMs are, however, too coarse for detailed medical explanations based on our CXR data. For this reason, we additionally applied the VarGrad method of Adebayo et al. (2018) for more fine-grained explanations.^3^ According to Hooker et al. (2019), VarGrad shows more faithful results than many other gradient-based XAI methods which the authors had compared to.

Apart from using a one-dimensional standard colormap to present the model’s explanations (e.g., as usually used with VarGrad), we extend the standard approach and propose a two-dimensional colormap (2Dcolormap) which linearly encodes the importance map returned by the specific XAI method with the pixel intensity of the original input image. This approach adds information on whether an indicated important image area corresponds to a dark or light region of the original image.

**Fig. 5:**
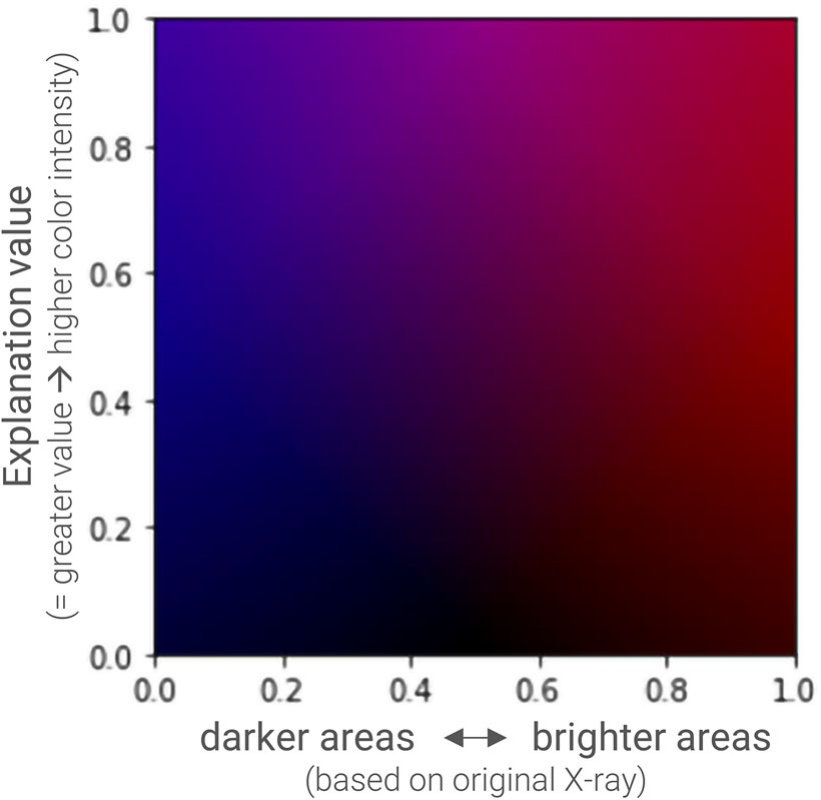
2D Colormap to visualize explanations, which linearly combines pixel importance predicted by the XAI Method (vertical axis) with pixel intensities of the input image (horizontal axis) (color figure online)

Figure 5 presents the coding of this colormap and Fig. 8 shows example explanations using this colormap. This extension ensures that one picture compiles all necessary information, and the user does not need to switch back and forth between examining the interpretation and the original X-ray (e.g., compare Evans et al. 2022).

## Evaluation Case Study: Generating Insights and Improving Models with XIL-ADR

Figure 3 shows that XIL-ADR can result in several loops/cycles, and each loop can generate new insights. This case study will describe the different loops we conducted in the *Implementation* stage of our medical case using XIL-ADR with experienced pneumologists and radiologists and how the result of one loop shaped the next loop. In effect, while this section describes the implementation phase of the medical case (conducted with XIL-ADR), it primarily serves as an exemplary evaluation case of our XIL-ADR method. As such, this description of the implementation does not focus on the results of each loop but on the developmental journey using XIL-ADR. The following subsections describe the XIL-ADR loops we conducted in the course of this implementation following the activities of the XIL-ADR cycles (see Fig. 3). In this context, we will also describe the intermediate artifacts (e.g., lessons learned, design requirements) that emerged from each activity. At the beginning of the XIL-ADR process, we describe the *Instantiation* of the model prototype (subsequent cycles start with *Refine Data and Retrain Model*). Next, we describe the activity of *Explaining, Visualizing and Inspecting* the model and its performance. This step forms the basis of the subsequent *Reflection* on the model explanations and performance, which leads to the generation of *Insights*. Finally, each loop results in the *Definition of XIL Strategies* for the subsequent loops. For an overview of the four conducted XIL-ADR cycles, the data basis, and their intermediate artifacts, refer to Table 2 at the end of this section.

### Loop 1

#### Model Instantiation

We first train the classifier as outlined in the last chapter and use the complete information on the X-ray for classification. This procedure is the standard approach, and the resulting classifier is (too) often the endpoint of a standard ML process. The disadvantages will become evident when we analyze the results with XAI and then continue with the IML part in the next loop.

#### Explain, Visualize and Inspect

Overall, the performance metrics indicate a particularly good model, with the model correctly predicting 98% of Normal, 83% of Viral Pneumonia, and 96% of Covid-19 cases. This result indicates that our model can distinguish lungs with pulmonary infiltrations from lungs without infiltrations (the patients can still be sick for other reasons) and can even distinguish standard viral pneumonia (without Covid-19 pneumonia) from the new Covid-19 pneumonia with extremely high accuracy. The model’s accuracy is 91.06%, precision is 93.64%, and recall is 92.53%. Such results would be highly beneficial, and a number of papers have shown similarly encouraging results on the basis of the same dataset.

#### Reflect

**Fig. 6:**
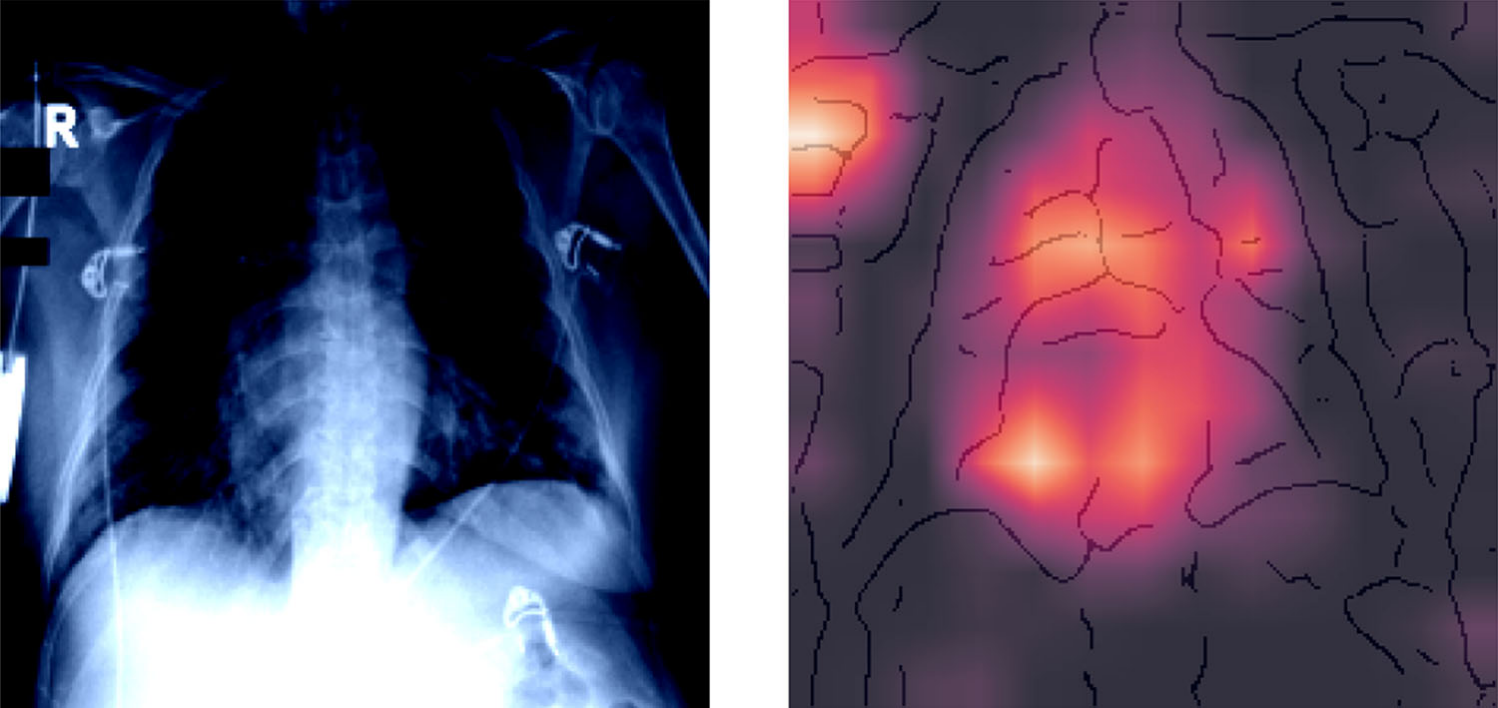
Loop 1 XAI. Original image on the left, XAI showing confounders with Grad-CAM method (Selvaraju et al. 2017) on an overlay of the edge-filtered image on the right (color figure online)

However, such a simple approach that is widely used in practice and research tends to overlook potential confounders. In addition, our loop 1 prototype suffers from such a potential problem as a detailed analysis of the explanation reveals: Fig. 6 presents a typical confounder on an X-ray of a Covid-19 patient. The visualization shows that the AI has detected predictive value in the ‘R’ on the left upper side and heavily uses it to predict the class of Covid-19 cases.

#### Insight

The ‘R’ on an X-ray typically marks the patient’s right side, but it cannot be found on all X-rays in our sample as it might have been left out or cropped, and the fonts used can also vary. Potentially newer X-ray machines include this marking more often, or hospitals/radiologists from a particular region hit hard by Covid-19 use machines that include this marking technique automatically. Furthermore, patients with Covid-19 often lie face down, which was found to improve lung recruitability^4^ (Pan et al. 2020), and hence it is more practical to place the ‘R’ on the detector (opposite to placing it on standing patients).

#### Define XIL Strategies

In the presence of such a bias in the data, the classification would work well on our test dataset, but it would fail to deliver satisfactory results on X-rays from out of sample, e.g., other regions that would not have the same bias. Therefore, forcing the AI to focus on regions that should be used for classification is mandatory. Indeed, it is possible to pre-process pictures accordingly, but it needs expertise to think about potential confounders. Furthermore, often the strenuous and unwelcome data work leads to incompleteness in this respect and thus also to corresponding cascading errors in the development process (Sambasivan et al. 2021).

Usually, the confounding effects are more challenging to detect than our illustrative example in Fig. 6 suggests, a problem we will exemplify in loop 2. To solve these problems, XIL-ADR and appropriate annotation tools allow experts to exclude problematic regions and can thereby force the algorithm to focus on different regions throughout the different replications of the process cycle. This fact would also allow us to evaluate whether certain other regions, like the heart, would create excellent predictors.

### Loop 2

#### Refine Data and Retrain Model

**Fig. 7:**
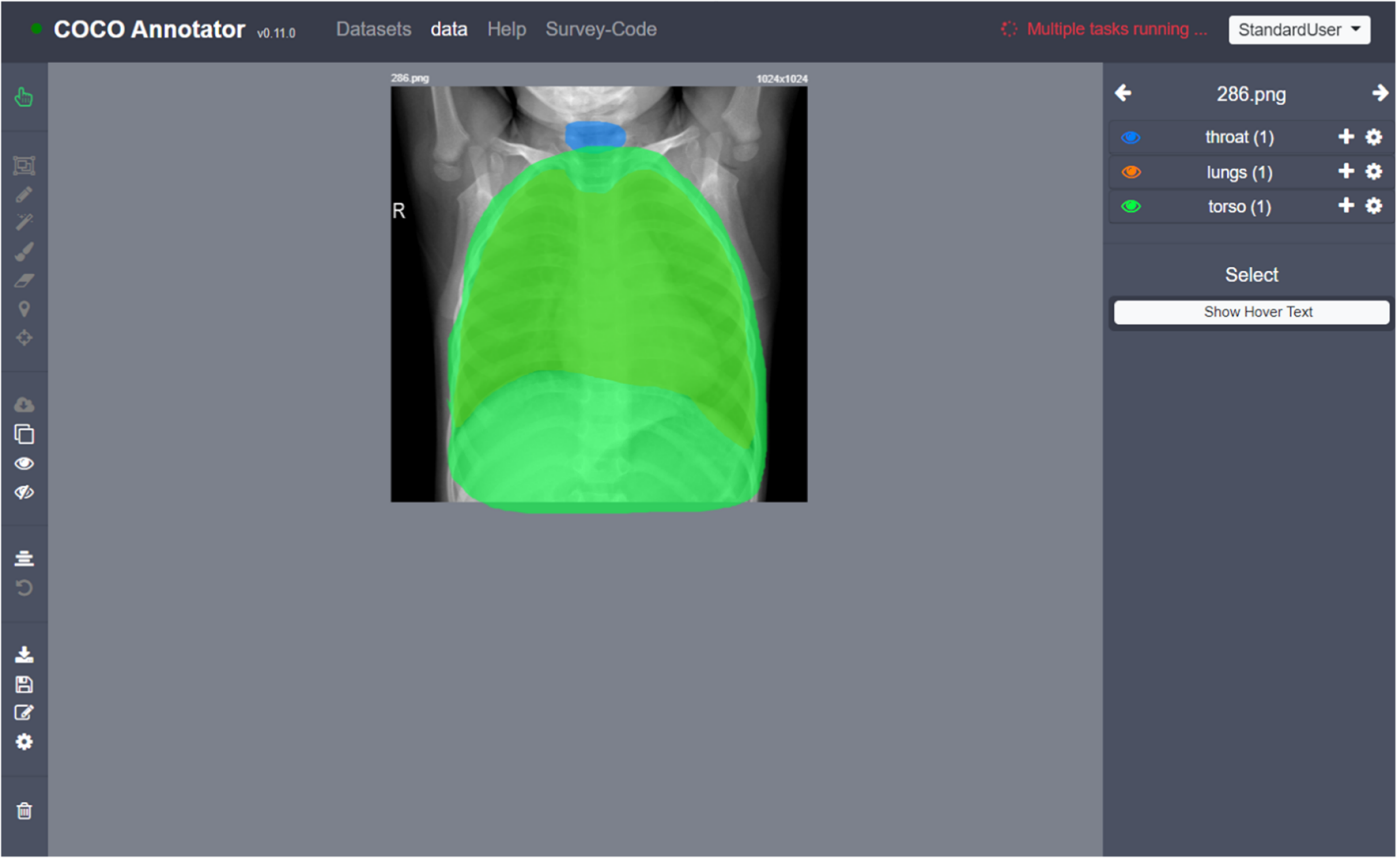
The user interface of the customized COCO Annotator used by the crowdworkers to annotate the images. Using the tools on the left on each X-ray, the areas for the throat, lungs, and torso were highlighted (color figure online)

To exclude obvious confounders, we compiled an instruction video and hired crowdworkers to annotate the dataset of nearly 3000 images. Figure 7 shows a tool based on the COCO Annotator (Brooks 2019) that we customized for our study. Based on the recommendation of our medical colleagues, we thereby instructed the crowdworkers to annotate three different regions: Torso, lungs, and throat, and merge them to create a fourth class which we call “Full”.

In loop 2, we trained another classifier that uses the annotation described in our technical methods section. Again, the model exhibits a superb goodness-of-fit (correctly predicted 99% of Normal, 91% of Viral Pneumonia, and 89% of Covid-19 cases). The accuracy of this model is 94.50%, precision is 96.30%, and recall is 92.93%.

#### Explain, Visualize and Inspect

**Fig. 8:**
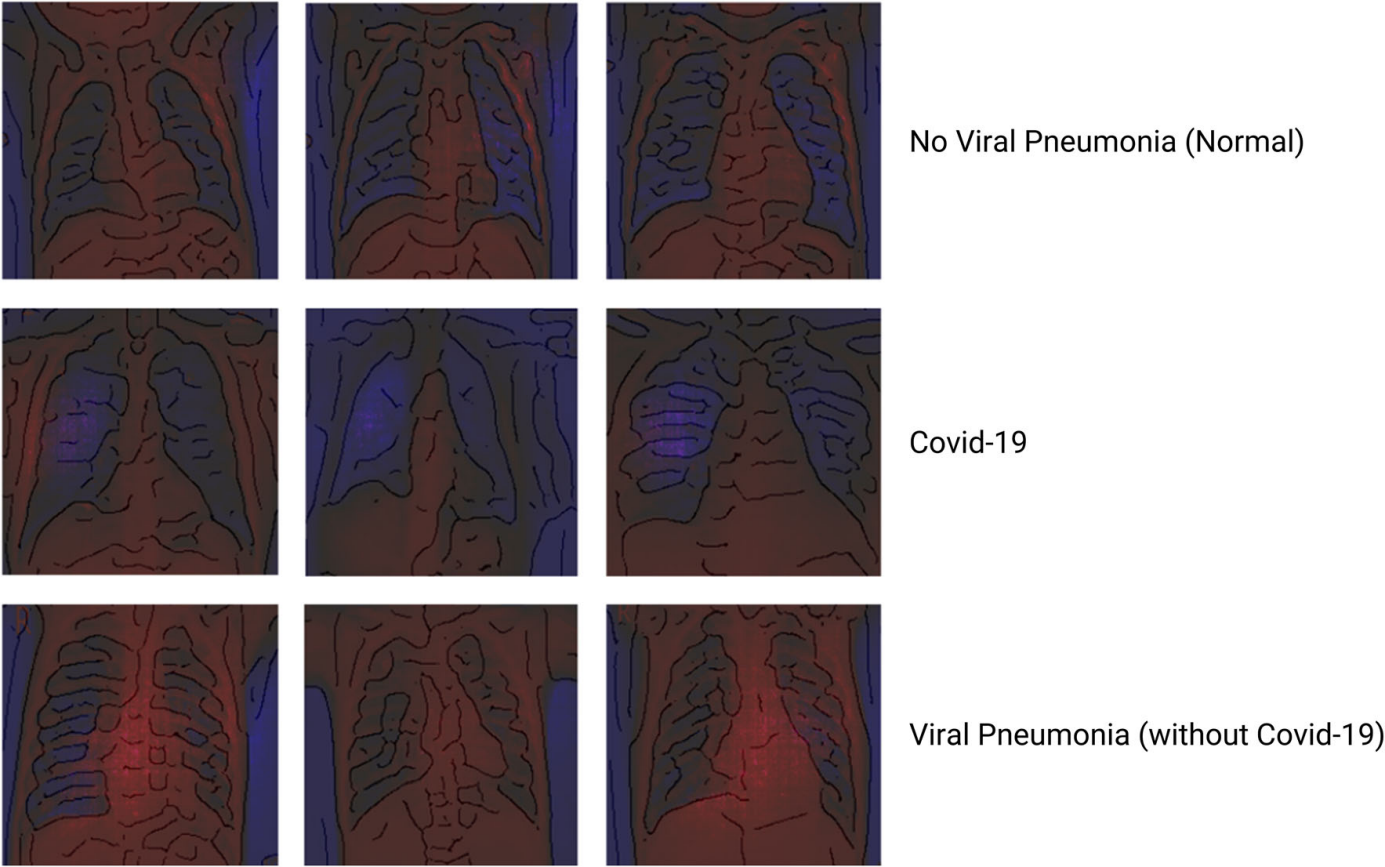
XAI showing Explanations for Classification in loop 2 (using our customized red-blue filter solution based on VarGrad (Adebayo et al. 2018)) (color figure online)

The results seemed to be very promising, and in a joint (virtual) session of three computer scientists, three information systems researchers, and four physicians (i.e., radiologists and pneumologists), we inspected the explanations, some of which are visualized in Fig. 8.

#### Reflect

Even for non-experts, it becomes evident through the XAI explanations that typical patterns on the X-rays lead to the classification. If non-experts can recognize these highlighted patterns on X-rays and then diagnose new diseases reliably, this would constitute a significant breakthrough. In this loop, we observe blue areas between the ribs for physiological subjects, indicating that these areas are important and that they are dark areas on the original X-ray image.

In contrast, we observe that patients with classical viral pneumonia have many red areas, indicating that bright areas on the original X-ray are of importance. In contrast to these two groups, we can identify Covid-19 cases by a cloud of blue and sometimes reddish points in the lung area.

#### Insight

While these results are promising for further inspection from the physicians, the explanations revealed another critical aspect that non-domain experts would not recognize so easily. Also, in our case, only the medical experts wondered about specific patterns. In some XAI images, we recognized that the algorithm marked areas around the shoulder as important. On closer inspection, these changes correspond with the developing skeletal features that are distinct for individual skeletal maturity stages of children and differentiate these images from adults. The closing of epiphyses, such as the humerus head epiphysis highlighted in Fig. 6, are examples of these fundamental anatomic differences between age groups. These predictors do not relate to the classification task or do not correlate with the ground truth indicating pneumonia or Covid-19-induced pneumonia.

This insight caused a further inspection of the X-ray image database, which revealed that the classes of viral pneumonia and normal cases indeed entirely consist of pediatric patients, i.e., children, while the Covid-19 cases are mixed and mainly consist of older adults. Further analysis of the data sources that contributed to the Kaggle winning dataset (Chowdhury et al. 2020) revealed that the XAI pointed to a hidden but very important confounder: The “Normal” and “Viral Pneumonia” CXR categories consist of images from the “Pediatric Bacterial and Viral Pneumonia Data Set” by Kermany et al. (2018)^5^ while the Covid-19 category consists of CXR of patients of various ages, yet primarily adult patients. In principle, this boils down to the classifier being able to distinguish between a CXR of an adult and a child. At least this biased data source partly explains the excellent classification of the holdout sample. In search of related papers that recognized this issue, we found only very few articles that address this problem and adjust their data selection process accordingly (e.g., Oh et al. 2020). Many other papers that rely on this dataset did not recognize and/or address this problem. In practice, we would observe another failed ML project where the standard training/test approach would lead to highly promising results but where the system would fail to deliver on new datasets in practice. Garcia Santa Cruz et al. (2021) also emphasize this issue in the available Covid-19 chest X-ray datasets and critique published papers blindly using those datasets for training ML models without accounting for potential sources of bias involved in the data.

#### Define XIL Strategies

After discussing this problem, we decided to pursue two different strategies. First, we excluded the Covid-19 cases from our dataset to see whether our algorithm could detect viral pneumonia in children. We describe this in loop 3a. Second, we curated a new dataset that does not implicitly lead to such biased results. We describe the insights about this fourth classifier in loop 3b.

### Loop 3a

#### Refine Data and Retrain Model

In this loop, we continue to work with the subsamples “Normal” and “Viral Pneumonia” of the Covid-19 database (Chowdhury et al. 2020) that were initially compiled by Kermany et al. (2018). It entirely consists of X-ray images from pediatric patients, and we can thus rule out the confounding problem from loop 2, and the identification of viral pneumonia in children alone is of importance. As of January 2023, a joint report^6^ from the American Academy of Pediatrics and the Children’s Hospital Association reported 15.24 million Covid-19 cases for children in the US registered from the onset of the pandemic. Furthermore, they reported in August 2021^7^ that between 1.6 and 3.5% of the total cumulated hospitalizations (of twenty-three states plus New York) due to Covid-19 were children.

#### Explain, Visualize and Inspect

Naturally, we use the created annotations to minimize the influence of potential confounders outside the area of interest. The accuracy of this model is 93.91%, precision is 94.13%, and recall is 93.91%.

#### Reflect

**Fig. 9:**
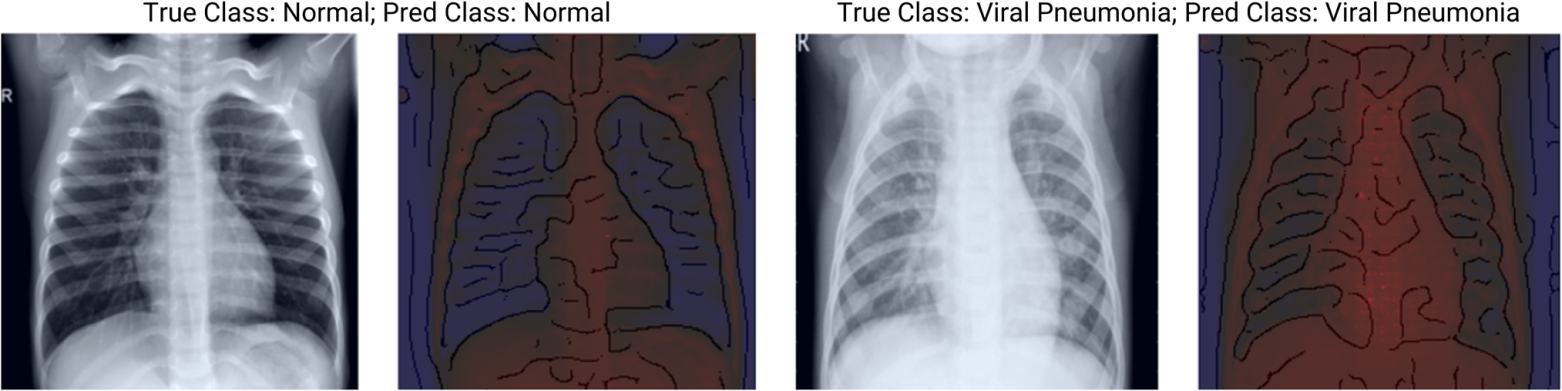
XAI showing Explanations for Classification in loop 3a (using our customized red-blue filter solution based on VarGrad (Adebayo et al. 2018)) (color figure online)

The algorithm detects healthy subjects in 98% of all cases and correctly identifies patients with viral pneumonia in 90% of all cases. Looking at the colored explanations in Fig. 9, we recognize some patterns from loop 2. The classifier uses bright parts of the X-ray in the lung to identify viral pneumonia. If these areas in the lung are darker, the algorithm classifies the case as normal.

#### Insight

Loop 3a is a very illustrative example but not as challenging as distinguishing between Covid-19 and classical viral pneumonia, and the results similar to loop 2 (cloud of blueish and reddish points in the XAI images) seem to be interesting. What may be problematic here, however, is that since patient metadata on the images is missing, patient leakage from the train into the test set may bias these results. This problem would also be the case for the Covid-19 dataset of loops 1 and 2.

#### Define XIL Strategies

We, therefore, started to curate a new dataset for training and testing from the “ChestX-ray14 database” by Wang et al. (2017), which involves a lot of work. We will analyze this dataset in loop 3b. In our quest to reduce bias while gaining an understanding of the model workings, we also compiled an additional independent test set from the database of the Hannover Medical School (Winther et al. 2020) for the Covid-19 class, which helps us to realize and exemplify the importance of independent patient holdout-testing.

### Loop 3b

#### Refine Data and Retrain Model

We use the “ChestX-ray14 database”^8^ by Wang et al. (2017) in this branch. This extensive database contains about 112,000 labeled CXR images of fourteen common thorax diseases, as well as CXR images without a diagnosis on any of the fourteen diseases, which are therefore labeled as “No Finding”. Due to the vast amount of CXR images and the trustworthy seal of the “National Institutes of Health”8, this dataset has been used extensively in the past three years in medical DL research and has enabled the pursuit of many research projects.

From this dataset, we utilize two categories to implement a three-class classifier: (1) we use the “No Finding” class to establish a baseline, and (2) we use the “Pneumonia” disease class to be able to distinguish our “Covid-19” class (from the Chowdhury et al. (2020) database) from other Pneumonia cases.^9^ In a multi-stage data preparation and selection process, we filter out (a) non-frontal images, (b) heavily cropped images, (c) images with poor resolution, (d) images with too prominent thoracic foreign material, and (e) images with an inappropriate windowing.^10^ We arrived at a higher-quality subset of the dataset through this process.

#### Explain, Visualize and Inspect

**Fig. 10:**
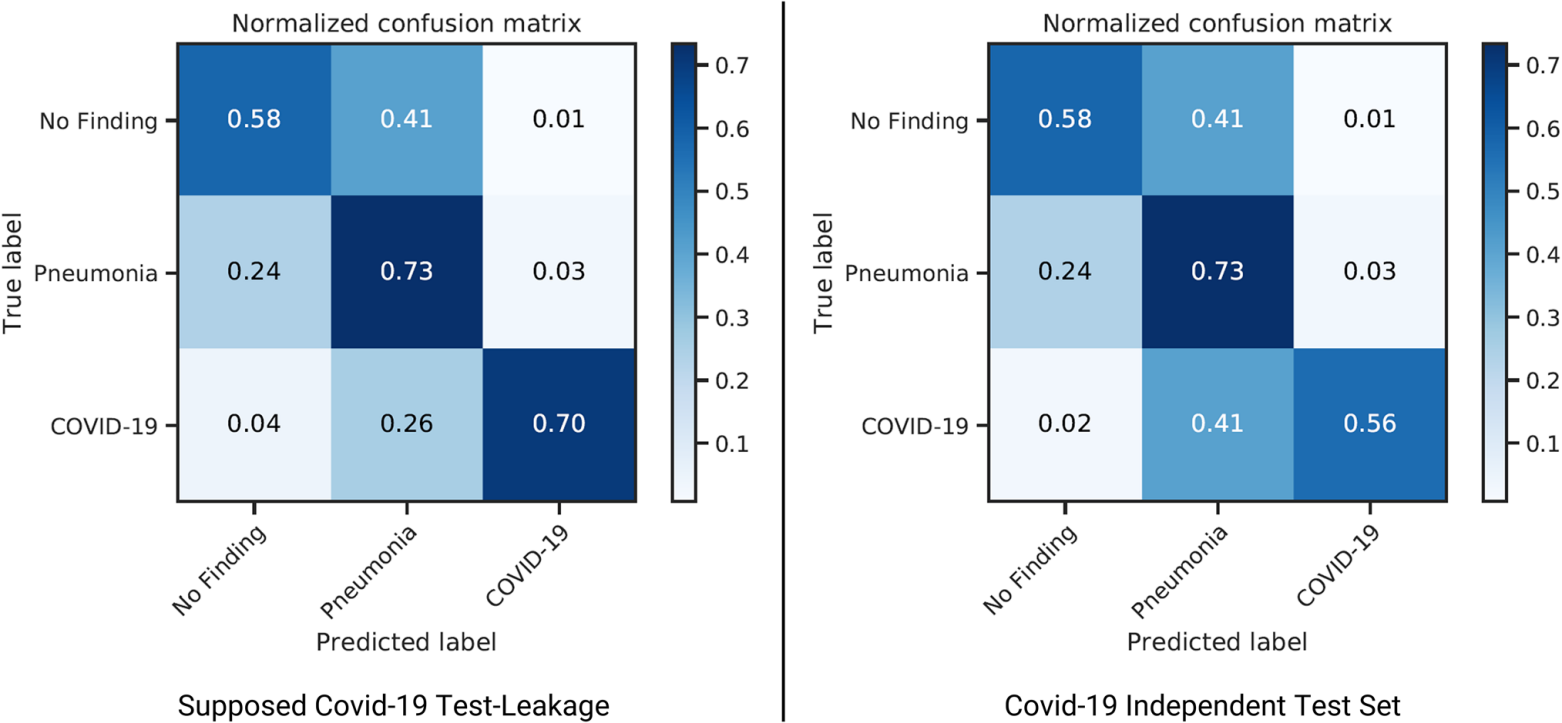
Confusion Matrices of loop 3b with Chowdhury et al. (2020) on the left and Winther et al. (2020) on the right

First, we realized that the new dataset made the classification much more challenging. It is easier for an algorithm to distinguish adults from children based on X-rays, but detecting patterns that explain diseases is harder. Based on the two testing strategies, i.e., testing on the Covid-19 images with suspected test leakage (Chowdhury et al. 2020) (left) and the independent Covid-19 dataset (Winther et al. 2020) (right), we arrive at the following confusion matrices depicted in Fig. 10. The accuracy of this loop’s model based on the original test set is 67.23%, precision is 72.27%, and recall is 67.23% (left). As the confusion matrix on the right in Fig. 10 shows, testing for the Covid-19 class with an independent test set revealed that the classifier appears to be struggling to discern other variants of Pneumonia from Covid-19-induced Pneumonia with out-of-sample data.

#### Reflect

The results are weaker than the ones reported by Mei et al. (2020) (75.9% sensitivity for the CNN), where CT images have been used, which have a much better resolution (but have other disadvantages) and where only two classes (Covid-19 vs. normal) were examined. Interestingly, as both confusion matrices indicate, most of the misclassification rate for Covid-19 falls on false-positive classifications for Pneumonia but not on No Finding. From a different perspective, this indicates that the classifier can recognize Covid-19 patients as ill patients quite well. We can also go one step further than Mei et al. (2020) and inspect the model using the XAI part of our proposed method. Again, the XAI often points to clouds of dots in the lung that seem to be a predictor of Covid-19.

#### Insight

One insight generated in loop 3b are the mentioned dot clouds. Potentially, these dot clouds could be indicators for vascular changes that might occur with Covid-19 infection (inflammatory, pulmonary reactions). Human experts usually find such details hard to detect by visually inspecting the original X-ray. The XAI part of the cycle can thus help humans by highlighting important areas.

At this point, a further curation of the dataset, training, and testing strategies (e.g., acquiring more images of distinct patient cohorts for testing, see Irvin et al. 2019) is necessary. Facing these major challenges, we therefore follow the ‘*Abort and Realign Strategy*’. Nevertheless, there may be potential in refining the model, as the confusion matrices indicate, and also labeling strategies to help the model find patterns that more effectively distinguish the Pneumonia class from the Covid-19 class. The future development process will be discussed in the “Outlook” section. Nevertheless, it became evident that XIL-ADR could help unveil confounding factors and thus contribute to the improvement of implementation requirements, as well as the generation of insights on the part of the human user.

### Outlook

After describing four loops of the XIL-ADR development process for our illustrative medical case study, the process ends here, as the challenges faced in this project require a deliberate and elaborate realignment of the strategy. Hence, the decision process at the end of the *Insights* activity resulted in the ‘*Abort and Realign Strategy*’.

Nevertheless, the produced models and intermediate artifacts have already contributed substantially to an increase in the knowledge base, ostensibly from an engineering perspective but also from a domain perspective; this result showcases a unique strength of XIL-ADR. The described loops have already revealed typical pitfalls and potential confounders that can heavily influence the resulting models in machine learning. Not only does the black-box problem make the results less reliable, but this problem also hampers scientific progress, although these new methods are so powerful and promising. This medical example shows simple, obvious confounding factors as well as more hidden confounders that only domain experts can recognize. The proposed process of XIL-ADR can mitigate two problems: First, it allows for identifying and removing potential confounders, and second, it allows us – as humans – to recognize patterns more easily and thus could potentially extend our knowledge base in the application domain. In this case, researchers from different disciplines will further engage in the human-machine-loop.

To ‘*Realign*’ the project with the defined goals, the next apparent steps for loop 4 include curating more datasets including metadata, which will help to ensure high-level patient-level splits. These quality splits are necessary to further test for systematic biases in system performance with the help of XAI (e.g., bias toward gender, age groups, or disease progression). Furthermore, we will annotate the newly curated training images to arrive at more refined results. In addition, data scientists can evaluate the true potential of more complex and highly acclaimed models with the help of XIL-ADR. For illustration purposes, we conclude here with the description of the first four cycles performed (see Table 2).

**Table 2:** Overview of the evaluation case with the four loops and their respective intermediate artifacts

Intermediate Artifacts	Loop 1	Loop 2	Loop 3a	Loop 3b
Initial dataset (n per class)	Normal: 1341 Viral Pneumonia: 1345 Covid-19: 219		Normal: 1341 Viral Pneumonia: 1345	No finding°: 594 Pneumonia°: 512 Covid-19: 219
Test dataset (n per class)	Normal: 336 Viral Pneumonia: 337 Covid-19: 54		Normal: 335 Viral Pneumonia: 337	No finding°: 141 Pneumonia°: 105 Covid-19: 47 *Independent testing: * No finding°: 141 Pneumonia°: 105 Covid-19*: 243
(Refined) Prototype	AlexNet Disease Classifier (ternary)	AlexNet Disease Classifier (ternary) with Annotations and Penalization (RRR)	AlexNet Disease Classifier (binary) with Annotations and Penalization (RRR)	AlexNet Disease Classifier (ternary)
Transparency-based evaluation criteria	Grad-CAM/VarGrad	VarGrad	VarGrad	VarGrad
Evaluation report	• Seemingly good performance • R’-type confounders found	• Seemingly good performance • Domain expertise confounders: Focus on skeletal regions	• Seemingly good performance • Possible train-test leakage may influence performance	• Seemingly average performance • Additional test set reveals generalization problems due to train image leakage
Insights	• ‘R’ typically marks the right side of a patient • Patients often lie face-down	• Systematic age differences in data confound the ability to learn	• Not as challenging as ternary classification	• Abnormality in images is detected quite well • Struggle to discern Covid-19 from other Pneumonia
XIL Strategies	• Penalize AI for focusing confounders (RRR) • Include citizen annotators • Definition of annotation regions • Teaching citizen annotators via video-instruction • Annotation of the whole dataset	• Curate new datasets	• Curation of a new dataset • Integration of radiologist demands in the data selection process (e.g., windowing)	• Collecting more patient data: additional test sets • Further curation through annotations

The majority of cases are from the “COVID-19 Radiography Database” by Chowdhury et al. (2020). For loop 3b, alternative classes from the “ChestX-ray14 database” (Wang et al. 2017) are indicated by a circle (°). The numbers for Wang et al. (2017) already represent case numbers after the multi-stage data preparation and selection process. The Covid-19 class for independently testing the model (Winther et al. 2020) is indicated by an asterisk (*)

## Discussion and Conclusion

This paper presented a novel situational ISDM called XIL-ADR. In engineering the method, we followed the approach of Goldkuhl and Karlsson (2020) by identifying the problem, theorizing, and engineering the method, before demonstrating and evaluating it in a healthcare development case. XIL-ADR is generalizable to iterative ML development-evaluation activities.

From an ADR perspective, XIL-ADR contributes to extending ADR (e.g., Baskerville et al. 2018, p. 365) such that research and organizational teams can use the methodology to conduct ADR more tailored to data science projects. In doing so, it proposes to shift the focus from an organizational *BIE* perspective (e.g., Sein et al. 2011) toward a more technical *BEI* perspective with five XIL-tailored activities and a focus on interventions toward the ML artifact, which helps to account for the technical intricacies of data science projects and especially ML-based system development.

Especially the combination of XAI and IML reinforces important parts of ADR (e.g., Mullarkey and Hevner 2019; Sein et al. 2011) in the development cycles and allows the participants to engage in *Reflection* and *Insight* activities, resulting in well-informed strategies for reengineering. For research, this may amount to better theoretical insights and to artifacts that are more refined. For practice, this presents the opportunity to achieve more efficient development cycles, with a higher potential to eliminate biases in ML-based IS, compared to other ISDM. Conclusively, XIL-ADR not only presents a valuable guideline for practitioners and researchers alike to uncover problematic issues with underlying ML strategies and data but also serves the purpose of more intriguingly supporting insight generation.

This article contributes in multiple ways: First, we show how XIL-ADR can lead to more meaningful models and help experts to validate existing recommendations and/or generate new insights in their application domain. Interactivity allows experts to force the algorithm to focus on specific areas in the data, and the algorithm can help the expert to better understand the data at hand by highlighting the patterns that determine its classification result. This information can either be used to assess the recommendations made by human experts^11^ or – in the best case – allow to identify new patterns that have not yet been recognized by human experts before (e.g., Teso and Hinz 2020). In that sense, the human-machine-loop can serve as a method to arrive at novel scientific insights, just like classical statistics helped us to better analyze and understand data in the past decades. We especially believe that the Information Systems discipline will play a crucial role in its development, as this is where human factors and expertise meet with technological developments. We used the example of imaging in medicine because it is very illustrative, but the methodology of XIL-ADR is, in general, also applicable to other types of data and other domains.

Second, we show that a simple training approach for ML models, which is currently widely used, can very often suffer from confounding factors that can heavily influence the results. In our illustrative case, confounders like tubes, marks, or texts (e.g., timestamps) on the X-ray can serve as predictors for classification, while this would not help us to predict new, out-of-sample cases. This problem can partly explain why so many AI classifiers with high accuracy on holdout test sets later fail to deliver in the real world. In tackling this problem and engineering XIL-ADR, we empower IS to fulfill an important mission and work against bias in ML systems (e.g., Kane et al. 2021, p. 375) in an effective manner. Thus, XIL-ADR also contributes to the stream of research on Human-AI Augmentation and directs the AI toward favorable outcomes (e.g., Teodorescu et al. 2021). As Lebovitz et al. (2021) state, many AI models are built quickly and yet achieve high performance metrics, but do so with lots of uncertainty about how these models using the underlying data perform so well, and with suspicions of flaws (e.g., regarding the data or model), which “*a diligent evaluation of such tools could have surfaced*” (Lebovitz et al. 2021, p. 1515). By combining XAI and IML, we can not only iteratively uncover but also eliminate such flaws and confounding effects, which otherwise would require excessive manual labor or cost-intensive processes. To combat confounders as part of one *XIL Strategy* in our evaluation case, we resorted to crowdsourcing guided by an instructional video, which helped code nearly 3000 X-rays manually with a customized annotation tool. For other use cases, depending on the type and amount of data, this annotation strategy may not warrant the expected results or may simply be too costly. As an alternative strategy, automated tools offer the chance to (semi-)automatically remove or mark confounders on images or other types of data. Furthermore, research and practice should experiment with the inclusion of diverse kinds of users in different roles, as well as different collaborative constellations to find effective and efficient strategies for specific ML tasks. From the viewpoint of data science, this is undoubtedly an important research field that could help XIL-ADR to become productive.

Third, we propose that task-specific visualization approaches to XAI may improve the activities of *Reflection* and *Insight* in XIL-ADR. Our approach, for example, extends current state-of-the-art visualization by incorporating the special characteristics of X-rays, the application domain medicine, and, in particular, radiology, which helped to analyze the domain data better. An iterative comparison to the constantly evolving stream of methods in XAI (e.g., Evans et al. 2022) could generate further extensions of such customized XAI solutions for the XIL-ADR process (e.g., XRAI by Kapishnikov et al. (2019), or Guided Integrated Gradients by Kapishnikov et al. (2021)). Recent research also suggests that even ostensibly insightful XAI methods should be subjected to vicious inspection of their fidelity (e.g., Adebayo et al. 2020; Tomsett et al. 2020). It would thus be recommended to try and assess different XAI methods (e.g., Teso and Kersting 2019) for the purpose of inspection, especially in the earlier cycles of XIL-ADR, and to compare their results regarding uncovering potential bias or confounding factors. For doing so, research on and methods of the assessment of causability of XAI (i.e., effectiveness in explaining) can prove of high value (Holzinger and Müller 2021).

Unlike CRISP-DM, XIL-ADR is embedded in a larger development context and benefits from prior and posterior defined activities in the ADR process, e.g., considering the business problem and its domain in the *Diagnosis* stage. Unlike other CRISP-DM adaptations, such as CRISP-ML (e.g., Studer et al. 2021), XIL-ADR explicitly incorporates XAI and IML methods and accounts for the data-driven intricacies of ML projects. Lastly, XIL-ADR – in contrast to traditional implementation phases in ADR – puts heavy emphasis on technical and data aspects while simultaneously focusing development team efforts on defining appropriate and effective strategies for subsequent ADR cycles.

In this paper, we proposed the methodology of XIL-ADR to enable research and practice to construct AI-based systems that are more reliable. We outlined in which way XIL-ADR presents an opportunity for improving machine-learning projects. We showed that ML projects can largely profit from the iterative XIL-ADR process by refining the model and the underlying data, training, and testing strategies, and also how such a process benefits from including multiple kinds of users. Lastly, through this iterative refinement of promising models, the full potential of AI for organizational learning may be unleashed.

Although XIL-ADR comprises some destructive constructionism which can be painful at times, we should not underestimate the potential of AI-based systems. Instead, we believe XIL-ADR helps to carefully grind the rough diamond of ML projects to arrive at actionable solutions that efficiently support organizational goals eventually.

All in all, by creating a virtuous circle of human and machine collaboration, we could not only make our systems smarter but also allow different kinds of users to enhance their knowledge in the focal application domain. This vision, however, requires potent AI systems that allow for interactivity, explainability, accountability, and a better understanding of this human-machine hybridity loop.

## Footnotes

1 For example, see https://www.covid19dataportal.org/ or https://www.kaggle.com/allen-institute-for-ai/CORD-19-research-challenge. Last Checked: 10.01.2023.

2 An updated version of the dataset is available on: https://www.kaggle.com/tawsifurrahman/covid19-radiography-database. We retrieved the version of the database in March 2020. Last checked: 10.01.2023.

3 As a foundation for the basic Grad-CAM and VarGrad method, we used the Python Captum Package: https://captum.ai. Last checked: 10.01.2023.

4 I.e., reopening collapsed areas of the lung. For more detailed explanations, please refer to Santos et al. (2015).

5 Available on: https://www.kaggle.com/paultimothymooney/chest-xray-pneumonia. Last checked: 10.01.2023.

6 For details, see the ‘State Data Report’ Version 1/5/2023: https://downloads.aap.org/AAP/PDF/AAP%20and%20CHA%20-%20Children%20and%20COVID-19%20State%20Data%20Report%201.5.23%20FINAL.pdf. Last checked: 10.01.2023.

7 ‘State Data Report’ Version 8/12/21: https://downloads.aap.org/AAP/PDF/AAP%20and%20CHA%20-%20Children%20and%20COVID-19%20State%20Data%20Report%208.12%20FINAL.pdf. Last checked: 10.01.2023.

8 Available on: https://nihcc.app.box.com/v/ChestXray-NIHCC. For details on NIH, see https://www.nih.gov/. Retrieved in June 2020. Both last checked: 10.01.2023.

9 In contrast to the dataset provided by Kermany et al. (2018), this dataset includes CXR images of patients of different ages and not only children. Furthermore, it must be noted that the “Pneumonia” disease category may not only contain viral pneumonia cases, but also bacterial pneumonia cases. The dataset does however not provide detailed information about the distribution of the cases that were caused by bacterial and viral pathogens.

10 Images with inappropriate windowing or contrast stretching are too dark or too bright compared to the standard level used in digital imaging. Our radiologists suggested excluding such images because of their low quality.

11 See e.g., recommendations by the German Radiological Society for Covid-19: https://www.drg.de/de-DE/5995/covid-19/. Last checked: 10.01.2023.
